# Impact of Two-Dimensional Particle Size Distribution on Estimation of Water Vapor Diffusivity in Micrometric Size Cellulose Particles

**DOI:** 10.3390/ma11091712

**Published:** 2018-09-13

**Authors:** Valentin Thoury-Monbrun, Hélène Angellier-Coussy, Valérie Guillard, David Legland, Sébastien Gaucel

**Affiliations:** 1 JRU IATE 1208—CIRAD/INRA/Montpellier Supagro/University of Montpellier, 2 Place Pierre Viala, Bat 31, F-34060 Montpellier, France; valentin@thoury.fr (V.T.-M.); helene.coussy@umontpellier.fr (H.A.-C.); valerie.guillard@umontpellier.fr (V.G.); 2 INRA, Biopolymères Interactions Assemblages (BIA), Rue de la Geraudière, F-44000 Nantes, France; david.legland@inra.fr

**Keywords:** cellulose, quartz crystal microbalance, particle size distribution, apparent diffusivity, water vapor

## Abstract

This work aims at assessing the impact of two-dimensional particle size distribution (2D-PSD) on the identification of water vapor diffusivity in micrometric size cellulose particles displaying a size aspect ratio lower than 2 and a cylindrical shape. First, different methodologies to obtain the two-dimensional (2D) particle size distribution (diameter versus length) were compared, based on image analysis. Then, experimental sorption kinetics were obtained by using a quartz crystal microbalance (QCM) coupled with a water vapor adsorption system. Diffusivity values were estimated when considering either the 2D-PSD or global descriptors, such as the mean or median diameter and length of particles. Results revealed that the use of an analytical approach when considering the 2D mean-PSD or the median-PSD was the most accurate way to get diffusivity values at the scale of particles in a polydisperse sample of cellulose particles. Following this approach, a water vapor apparent diffusivity of 3.1 × 10^−12^ ± 2.3 × 10^−12^ m^2^·s^−1^ was found for the considered cellulose sample. Neglecting PSD in diffusivity estimation led to an underestimation of a factor of 2. This procedure could be extended for all the polydisperse samples in order to have an accurate estimation of water vapor diffusivity at the scale of single particles.

## 1. Introduction

The use of lignocellulosic and cellulose fillers in biocomposite materials is exponentially increasing this last ten years, which is mainly due to their worldwide availability at low cost, renewability and fully biodegradability, and ability to develop new functionalities. They present a real potential for different sectors of application, including automotive, agriculture, building and packaging [1,2,3,4]. Diffusion of gases and vapors is one of the key properties of materials aiming at being used in applications that are based on mass transfers properties, e.g., membrane or food packaging [5,6,7,8]. The water vapor is the major migrant of interest, since its diffusion in lignocellulosic fillers strongly impacts (i) the overall structural and functional stability of biocomposites in a moist environment, as well as (ii) their water vapor permeability, which governs many food degradation reactions. Hydrogen bonds that can be formed between hydroxyl groups of cellulose are very strong, resulting to ordered structures, i.e., crystalline regions. On the opposite, amorphous regions result from the poor stabilization of hydroxyl groups through hydrogen bonding [9,10,11]. The amorphous phase is thus the greatest contributor to the establishment of interactions between cellulose and water due to the easy accessibility of hydroxyl groups [12,13,14,15]. The crystallinity of cellulose is thus known to impact the water vapor sorption process: the water vapor sorption increases with the reduction of crystallinity [16,17]. Contrary to the water sorption, the diffusivity value cannot be directly measured. For this purpose, a gradient of concentration or partial pressure must be imposed to the sample, and the subsequent mass transfer must be measured against time, such as, for example, by measuring water vapor sorption kinetic. The diffusivity is then identified by fitting a mathematical model that is based on Fick’s second law to the experimental sorption kinetics. The diffusivity value is the one that minimizes the sum of squared error between the two datasets. Water vapor sorption kinetics could be obtained by gravimetrical methods, either discrete methods, e.g., isopiestic methods, or continuous ones, e.g., dynamic vapor sorption (DVS), intelligent gravimetrical analyzer (IGA), thermogravimetric analysis (TGA), or quartz crystal microbalance (QCM). While a numerical approach is often required for complex situations, analytical solutions exist for simple geometries and well-defined initial and boundary conditions [18]. These analytical solutions are strongly related to the geometry of the system. Consequently, a lack of knowledge or too strong hypotheses on the system geometry can lead to a poor, even false, estimation of the diffusivity. It is worth noting that the majority of the studies dealing with the estimation of water vapor diffusivity in cellulose-based materials are focusing on long fibers with a dimension higher than 1 cm, such as flax, jute, or agave [19,20]. Diffusivity was also estimated from associated fibers, such as films or papers [5], or samples of raw biomasses, such as wheat straw, raffia, or wood [7,21,22]. Only one paper has been recently published on the estimation of water vapor diffusivity in micrometric size cellulose particles [23]. However, global descriptors of particle morphology have been used to estimate diffusivity, without considering the impact of the whole size distribution.

In the case of sample powder corresponding to a population of dispersed particles, the sample shape is generally deduced from microscopic observations and detailed morphological parameters require image analysis. As regards to the dimensions that have to be used as input parameters in models, authors usually consider global descriptors for the size distribution e.g., median or mean, which implies to assume a homogeneous population of identical particles. Dealing with the estimation of water vapor diffusivity in particles with shape close to spheres or short cylinders, previous studies either considered the mean particle diameter in surface to assess water diffusion in maltodextrin powder [24], used the weight average radius that was evaluated by optical microscopy observations in the case of agave fibers [19] or used medians average in volume of diameter and length assessed by image analysis in the case of cylindrical cellulose particles [23]. Some authors demonstrated the importance of considering a dimensional particle size distribution (PSD) instead of a global descriptor to evaluate mass transfer properties in heterogeneous size particles [25,26]. As an example, Carta et al. [25] incorporated PSD by using a dimension radius distribution to predict adsorption phenomena of the protein chymotrypsinogen on SP-Sepharose-FF and compared this approach to results obtained by using an average radius value (r = 47.7 µm). They showed that PSD had a significant effect on predicted diffusivity absorption phenomena, this effect was more important for asymmetrical PSD with a better correlation between the experimental curves and predicted curves. Kaczmarski et al. [26] compared the use of dimensional PSD and mean particle diameter to describe protein adsorption in an expanded bed of protein. They concluded that the two approaches led to identical results for small particles (average diameter = 50 µm) while the use of PSD must be preferred in the case of bigger particles (average diameter = 210 µm). Angellier-Coussy et al. [27] also demonstrated the interest of considering the size aspect ratio values (one dimension) to predict the mass transfer properties in nanocomposites. It is worth noting that all of these studies only considered one-dimensional PSD focusing on variation of one size descriptor, while others are fixed, e.g., studying the effect of radius change on cylinder with a constant length.

In this context, the present study aims at assessing for the first time the impact of two-dimensional (2D) particle size distribution on the identification of water vapor diffusivity for micrometric size cellulose particles. First, a morphological analysis was performed on cellulose particles presenting a size aspect ratio (ratio between the length and the diameter) that was lower than 2. Different methodologies to obtain the 2D particle size (radius and length) distributions were compared, based on image analysis. Experimental sorption kinetics were obtained by using quartz crystal microbalance (QCM), coupled with an adsorption system. From these kinetics, water vapor diffusivities were estimated for a population of cylindrical particles, taking into account the 2D PSD, or alternatively by considering global descriptors, such as mean or median diameter and length of the cylinder. Finally, the necessity of considering the whole 2D particle size distribution instead of global descriptors was discussed.

## 2. Materials and Methods

### 2.1. Materials

Highly pure (99.5%) cellulose supplied by J. Rettenmaier & Söhne GmbH + Co KG (Rosenberg, Baden-Württemberg, Germany under the reference Arbocel@ BE 600-10 Tg was used in the study. According to the supplier, particles were characterized by an average length of 18 µm, an average thickness of 15 µm, and an apparent bulk density between 0.23–0.30 g·cm^−3^. Cellulose was quasi-amorphous, with crystallinity lower than 5%. Absolute ethanol (purity of 99.9%), which was used for the preparation of cellulose particles suspension, was provided from Meridis (Montpellier, Occitanie, France). Gold sensor crystals with a nominal frequency equal to 6 MHz were purchased from Neyco (Vanves, Île-de-France, France).

### 2.2. Methods

#### 2.2.1. Particle Morphology

**Optical microscopy.** Images of particles were obtained by using a macroscope Nikon Multizoom AZ100 (Tokyo, Kantô, Japan) equipped with a RGB DS-Ri1 camera (Tokyo, Kantô, Japan) in light transmission mode. 40 µL of a suspension of cellulose in absolute ethanol at a concentration of 0.35 g L^−1^ was dropped on a microscope glass slide. Ethanol was then evaporated under vacuum. The magnification was set to ×25 by adjusting the lens AZ-Plan at ×5 and setting the optical zoom to ×5. To avoid unclear particles, a vertical displacement (z axis) was added by seven steps of 4 µm. Mosaic images were assembled by reconstructing 5 × 5 images. Nikon software NIS-Elements (Tokyo, Kantô, Japon) operating with the Multizoom AZ100 system was used to combine these images. This microscope allowed for distinguishing all particles of area greater than five pixels, i.e., greater than 4.2 µm^2^.

**Image analysis.** Mosaic images obtained by optical microscopy were analyzed by using the ImageJ software (North Bethesda, Maryland, United States). Major axis (length of the inertia ellipse) and minor axis (height of the inertia ellipse) were determined for each particle. Image processing was performed on an increasing number of particles until that the cumulative average of the determined indicator (major axis or minor axis) was stable. Three different mosaic images were used containing a total of 3300 particles. Particles were assumed to have cylindrical shapes. The cylinder volumes were obtained from the major and minor axis values.

**Scanning electron microscopy (SEM).** SEM microscopy images were acquired with a high-resolution field emission gun Hitachi SEM S-4800 (Tokyo, Kantô, Japan) with an acceleration voltage of 2 kV. Prior to SEM analysis, cellulose particles were coated with a thin layer of platinum in order to avoid sample charging anomalies. This observation allows for obtaining qualitative information on the shape of particle.

#### 2.2.2. Water Vapor Sorption Kinetics

**Quartz preparation.** The deposition of cellulose on the quartz crystal substrate was executed in two steps. First, a drop of a suspension of cellulose in ethanol (c = 0.07 g·L^−1^) was deposited on the active area of the quartz. Then, ethanol was evaporated under vacuum to keep on quartz only the dispersed cellulose particles. The initial mass of cellulose ranged from 1 to 1.5 µg. A more detailed description of this preparation was given in the study of Thoury-Monbrun et al. [23]. Knowing the total mass of deposited cellulose and that the mass of one particle of cellulose was around 5.7 × 10^−4^ µg (value estimated from the true density of cellulose particles and the volume of one particle), it was calculated that around 2000 particles were deposited on each quartz substrate. 

**QCM measurement.** Water vapor sorption kinetics were performed while using a QCM equipment Maxtek TM-400 (Santa Fe Springs, California, United States) coupled with an adsorption system. This apparatus allowed for measuring the frequency variation Δf induced by cellulose water vapor uptake according to time. This frequency variation is linked to the mass variation Δm according to the Sauerbrey equation (Equation (1)) where $f_{q}$ is the nominal frequency (6 MHz) of the quartz not loaded, C an equipment constant (1.65 × 10^5^ cm·s^−1^), $\rho_{q}$ = 2.65 g·cm^−3^ the quartz density, and S the active area of the quartz (0.5 cm^2^). In this study, Δm corresponded to the water vapor uptake (µg) of cellulose particles.

(1) $$\Delta f=-\frac{f_{q}^{2}\times \Delta m}{C\times \rho_{q}\times S}$$

The quartz substrate loaded with cellulose particles was placed in a closed chamber where the pressure of water vapor was able to vary from vacuum up to 31.7 mbar (3170 Pa), which corresponded to the maximum pressure of water vapor at 80% of relative humidity and 25 °C. Water vapor uptake was calculated from Equation (1), where m is the water vapor that was sorbed by the cellulose particles (Equation (2)).

(2) $$m=-\frac{\Delta f}{f_{q}^{2}}C\times \rho_{q}\times S$$

The sample was dried at 25 °C during 30 min under vacuum before starting the experiment. Initial mass of cellulose deposited on quartz was determined by measuring the difference of resonance frequency between pristine quartz and loaded quartz. Eight successive steps of constant relative humidity (RH) from 0 to 80% RH were programed to obtain sorption kinetic (steps of 10 min each). Six replicates were done on six different quartz. 

#### 2.2.3. Modeling

Cellulose particles were assumed to have a cylindrical shape. A finite cylinder is the intersection of an infinite cylinder of the same radius and an orthogonal infinite plane sheet of the same height. Consequently, the analytical solution for the finite cylinder was obtained by combining the solutions that were provided for an infinite cylinder and an infinite plane sheet [18]. This so-called product of solutions was described by Matthews and Walker [28]. Two models were considered to simulate water vapor diffusion in the cylinder-shaped cellulose particles: (i) the first one assumed a homogeneous population of cylinders, i.e., displaying identical dimensions, while (ii) the second one described the same phenomenon in a heterogeneous population characterized by a 2D PSD. Diffusion was assumed to be isotropic and independent of position and time in cellulose particles.

**Diffusivity in a single cylindrical particle**. Let focus on a single cylindrical cellulose particle of radius R (m) and length L (m) described by r ∈ [0, R] and z ∈ [−L/2,L/2]. Diffusion equation in a finite cylinder geometry for a compound of concentration D(t,r,z) is defined by Equation (3), where t is the time (s), r is the radial position (m), z is the axial position (m), and D is the diffusivity (m^2^·s^−1^).

(3) $$\frac{\partial C}{\partial t}\left(t,r,z\right)=D\left(\frac{1}{r}\frac{\partial C}{\partial r}+\frac{\partial^{2}C}{\partial r^{2}}+\frac{\partial^{2}C}{\partial z^{2}}\right)$$

Assumptions of uniform initial concentration $C_{0}$ and constant and uniform boundary concentration $C_{\infty }$ were also done, as formalized in Equation (4). It was assumed that the particles were perfect rigid cylinders deposited according to their length, allowing for concluding that the contact surface was zero.

(4) $$C\left(0,r,z\right)=C_{0}\;\forall \;r\in \left[0,R\right]\;\mathrm{and}\;\forall \;z\in \left[\frac{-L}{2},\;\frac{L}{2}\right]\;C\left(t,r=R,z\right)=C_{\infty }\;\forall \;t\;\geq 0\;\mathrm{and}\;\forall \;z\in \left[\frac{-L}{2},\;\frac{L}{2}\right]\;C\left(t,r,z=\pm \;\frac{L}{2}\right)=C_{\infty }\;\forall \;t\;\geq 0\;\mathrm{and}\;\forall \;r\in \left[0,R\right]$$

Equation (5) presents the product of the solutions for the radial part and the axial part of the diffusion to obtain the analytical solution for a finite cylinder where $M_{t}$ and $M_{\infty }$ are, respectively, the mass of water vapor sorbed in the cylinder at time t and at infinite time and $q_{n}$ positive roots of Equation (6) where $J_{0}$ is the Bessel function of the first kind of order 0. Details on this analytical solution can be found in Thoury-Monbrun et al. [23].

(5) $$\frac{M_{t}\left(t,D,L,R\right)}{M_{\infty }}=1-\left(1-4\overset{\infty }{\underset{n=1}{\sum }}\frac{\exp\left(-{\mathrm{Dq}}_{n}^{2}t\right)}{R^{2}{\;q}_{n}^{2}}\right)\times \left(1-8\overset{\infty }{\underset{n=0}{\sum }}\frac{1}{{\left(2n+1\right)}^{2}\pi^{2}}\exp\left(\frac{-D{\left(2n+1\right)}^{2}\pi^{2}t}{L^{2}}\right)\right)$$

(6) $$J_{0}\left({R\;q}_{n}\right)=0$$

**Apparent diffusivity in a homogeneous population of cylindrical particles.** In this special case, all cylinders are assumed to have the same radius and length. As they are also supposed to be well-dispersed, i.e., without any contact between particles, all the cylinders have the same behavior regarding sorption of any compound. Finally, Equation (5) stays valid, where $M_{t}$ and $M_{\infty }$ become the mass of water vapor sorbed in the whole population of cylinders at time t and the corresponding value for an infinite time. This model will be used when considering global size descriptors, i.e., median values of radius and length, to estimate water vapor diffusivity in these cellulose particles.

**Apparent diffusivity in a heterogeneous population of cylindrical particles.** If dealing with a heterogeneous population of cylindrical particles, Equation (5) must be adapted to integrate the population size distribution. Assumption was made that all particles were constituted of the same homogeneous material, whatever their dimensions, so that the diffusivity did not depend on the particle size. It was supposed that the population was structured in N classes characterized by dimensions ($L_{i}$, $R_{i}$) with $L_{i}$ and $R_{i}$ the length and the radius of cylinders in class i, respectively. In other terms, all the particles in a given class were supposed to have the same dimensions, so that Equation (5) could be applied. The analytical solution becomes Equation (7) where $M_{t}^{\mathrm{het}}$ and $M_{t}^{i}$ are the mass of compound sorbed at time t in the whole population of particles and in the particles of class i, respectively, $M_{t}^{i}$ being calculated from Equation (5).

(7) $$M_{t}^{\mathrm{het}}=\overset{N}{\underset{i=1}{\sum }}M_{t}^{i}\left(t,D,L_{i},R_{i}\right)$$

At infinite time, Equation (7) becomes Equation (8), where $M_{\infty }^{\mathrm{het}}$ and $M_{\infty }^{i}$ are, respectively, the mass uptake of compound at equilibrium for the total population of particles and for the particles of class i, respectively.

(8) $$M_{\infty }^{\mathrm{het}}=\overset{N}{\underset{i=1}{\sum }}M_{\infty }^{i}$$

It is worth noting that $M_{\infty }^{i}$ is experimentally inaccessible. Nevertheless, remembering that all the particles are constituted of the same material and that the equilibrium is reached, it could be considered that the mass uptake $M_{\infty }^{i}$ depended only on the mass or volume of cellulose particles in class i. Consequently, $M_{\infty }^{i}$ is proportional to $M_{\infty }^{\mathrm{het}}$ through the mass fraction $w_{i}$ of particles in class i, as described in Equation (9).

(9) $$M_{\infty }^{i}=w_{i}\cdot M_{\infty }^{\mathrm{het}}$$

Finally, the analytical solution useful for prediction of $M_{\infty }^{het}$ rewrites as Equation (10).

(10) $$\;M_{t}^{\mathrm{het}}=\overset{N}{\underset{i=1}{\sum }}w_{i}\cdot M_{\infty }^{\mathrm{het}}\cdot \left[1-\left(1-4\overset{\infty }{\underset{n=1}{\sum }}\frac{\exp\left(-{\mathrm{Dq}}_{n}^{2}t\right)}{R_{i}^{2}{\;q}_{n}^{2}}\right)\cdot \left(1-8\overset{\infty }{\underset{n=0}{\sum }}\frac{1}{\left(2n+1\right){}^{2}\pi {}^{2}}\exp\left(\frac{-D\left(2n+1\right){}^{2}\pi {}^{2}t}{L_{i}{}^{2}}\right)\right)\right]$$

A special application case of this equation concerns a heterogeneous population of N cylindric particles. Indeed, if N is sufficiently small to allow numerical computation, Equation (10) can be applied, with $R_{i}$, $L_{i}$, and $w_{i}$ the radius, the length and the mass fraction of particle i, respectively.

**Two-dimensional size distribution.** In order to be considered in the estimation of the apparent diffusivity, the population of particles must be translated in terms of its population size distribution. The following methodology was applied. The population was sorted according to the diameter and length of particles. Five squared classes of decreasing side were used: 20 µm (18 classes), 10 µm (72 classes), 5 µm (288 classes), 2.5 µm (1152 classes), and 1 µm (7200 classes). For each class, the volume fraction linked to the mass fraction of particles was calculated. The sub-population of each class was then assumed to be a homogenous population of diameter and length, which were determined by using the three following indicators: center, mean and median. The resulting 2D particle size distributions (PSD) were then used in Equation (10) to estimate apparent diffusivity, and they were denominated as PSD-center, PSD-mean, and PSD-median respectively.

**Parameters estimation.** Numerical simulations were realized by using the Matlab^®^ (Natick, MA, USA) software and the lsqnonlin function to estimate two parameters: apparent diffusivity D of water vapor in cellulose and equilibrium mass of sorbed water vapor $M_{\infty }$. Parameter estimation was a non-linear least squares problem and consisted in minimizing the root mean squared error (RMSE) between experimental mass uptake $M_{\exp}$ and predicted mass uptake $M_{\mathrm{sim}}$, as in Equation (11).

(11) $$\mathrm{RMSE}=\sqrt{\frac{\sum_{i=1}^{N}{\left(M_{\mathrm{sim}}\left(t_{i}\right)-M_{\exp}\left(t_{i}\right)\right)}^{2}}{N}}$$

$M_{\mathrm{sim}}$ was computed by using alternatively Equation (10) when considering the PSD, and Equation (5), when considering only global descriptors for the size of the particles. Here N denotes the number of experimental data points.

#### 2.2.4. Statistical Analysis

Microsoft XLstat software 2008 (Île-de-France, Paris, France) was used for data processing. One-way analysis of variance, followed by non-parametric Friedman test was carried out to test the significance differences of diffusivity. The significance level was set to *p* < 0.05.

## 3. Results and Discussion

### 3.1. Morphological Analysis

**Figure 1:**
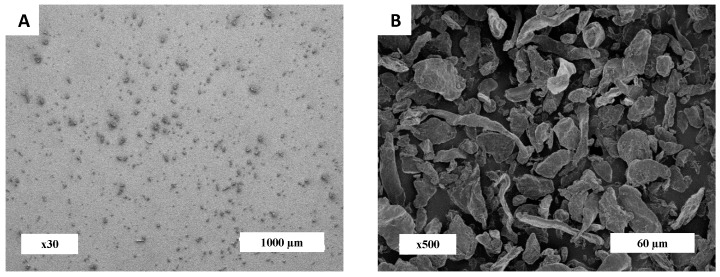
Scanning electron microscopy (SEM) images of cellulose particles of magnitude (**A**) ×30 and (**B**) ×500.

The morphology of cellulose particles was qualitatively assessed by SEM observations (Figure 1). A heterogenous population was highlighted, with the presence of small and round particles with dimensions lower than 10 µm, thin particles with diameter lower than 5 µm and also various lengths and bigger particles with dimensions higher than 40 µm. All of the particles were assumed to be cylindrical, with a major axis corresponding the length and a minor axis corresponding to the diameter. The distribution of these two dimensions was then quantified.

**Figure 2:**
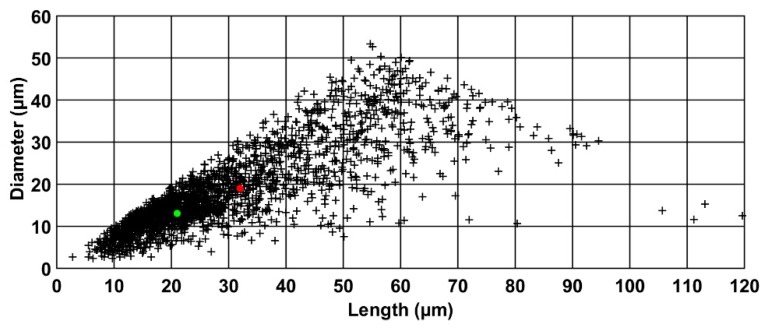
Scatter plot of the length according to the diameter of cellulose particles (red point = median value in volume, green point = mean value in volume).

Figure 2 illustrates a two-dimension (2D) repartition of the population of particles according to their diameter (µm) and their length (µm). For this example, the population was structured into 288 particles classes of size 10 µm ×10 µm (12 classes of 10 µm for length ×6 classes of 10 µm for diameter), with only 52 classes actually containing particles. The scatter plot showed an irregular shape with a high dispersion of both the length and the diameter. Cellulose morphology presented a high concentration of small particles with length ranging in the interval [5, 35] and diameter in the interval [5, 20], with 67% of particles present in this region. Larger particles, i.e., with a length higher than 50 µm, represented 6% of the population. Although they represented a lower number of particles than the small ones, these large particles had a non-negligible total volume and they must be thus considered when dealing with mass transfer properties.

**Figure 3:**
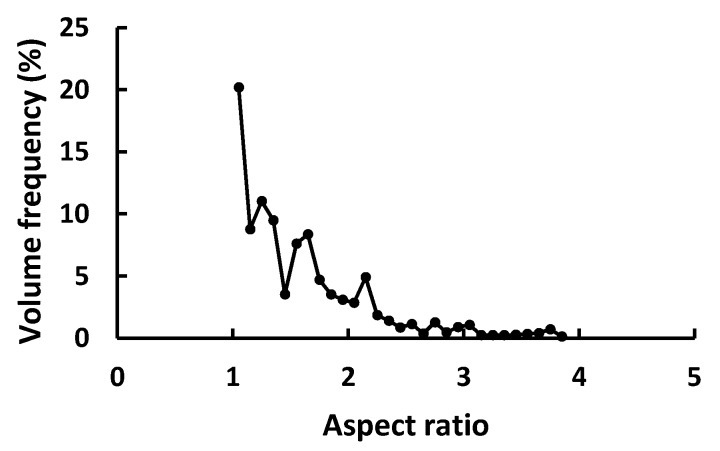
Volume frequency distribution of aspect ratio of cellulose particles.

In order to globally describe the PSD, the mean and median parameters in volume were computed. Due to the size dispersion of the population, a significant difference was observed between mean values (mean length of 21 µm and mean diameter of 13 µm), and median values (median length of 32 µm and median diameter of 19 µm). However, it is worth noting that the two sets of valued led to the same size aspect ratio (also called elongation factor, which corresponds to the length to diameter ratio), i.e., close to 1.6. Figure 3 presented the frequency of aspect ratio distribution. Particle size heterogeneity could be clearly deduced from this distribution. A large fraction of particles (around 70 vol%) had an elongation factor lower than 1.5, while 15% had an elongation factor superior than 2.

**Figure 4:**
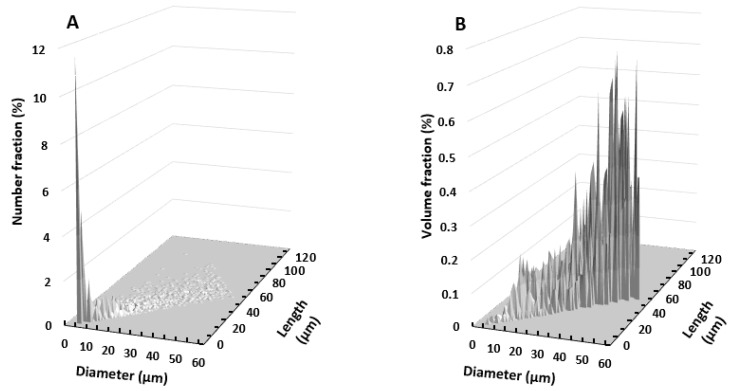
Two-dimensional (2D) particles size distribution in (**A**) number and (**B**) volume.

Joint distributions of length and diameters are presented in Figure 4 using 2D histogram representation. A bin size of 1 µm was used for both length and diameter axes. Two different distributions were considered: number-weighted distribution and volume-weighted distribution. Depending on the choice of particle representation, the size dispersion was different. The number distribution resulted in only few but important peaks located at small diameter and length and could lead to the biased conclusion that the population is homogenous (Figure 4A). The endorsed risk by considering the number distribution would be to neglect the less present but much larger particles, which may have a high contribution on mass transfer. In contrast, the volume distribution showed a more scattered shape, favoring the impact of large particles (Figure 4B). It is therefore essential to choose the adapted representation according to the studied properties. Regarding the characterization of mass transfer properties, which constitutes the core of this study, the volume distribution should be preferred to the number distribution. Indeed, mass transfer in these particles is affected by their mass or volume, with both being related to their density. The influence of the 2D volume size dispersity on the estimation of water vapor diffusivity is presented in the following section.

### 3.2. Water Vapor Diffusivity

**Figure 5:**
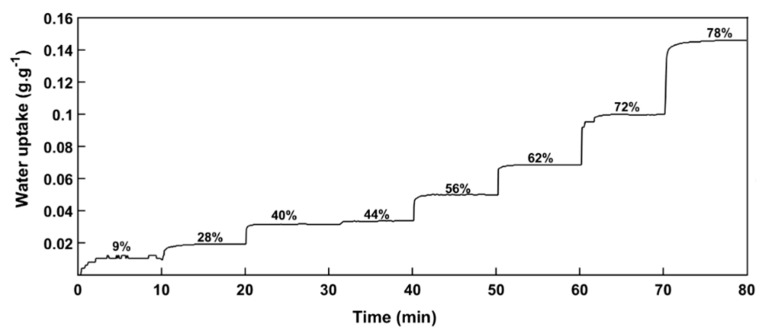
Water vapor sorption kinetic of cellulose particles measured by quartz crystal microbalance (QCM). The mentioned percentages correspond to the successive relative humidity steps.

Water vapor diffusivity was calculated at different steps of relative humidity (RH) by fitting sorption kinetic curves (Figure 5) with Equation (5) or Equation (10), depending on whether particle size distribution or a global indicator was used. As previously observed [23], the RH steps were difficult to adjust. That is why RH step was not incremented by constant steps. As compared to other sorption equipment, QCM allows for measuring sorption kinetics on a very small quantity of sample, i.e., between 1 and 1.5 µg. As the particles were well-dispersed on the quartz, it was concluded that obtained results were close to the behavior of a single particle. Additionally, due to this small amount of cellulose for each sample, this method is very fast with a water vapor equilibrium being reached in less than 3 min for each step.

**Figure 6:**
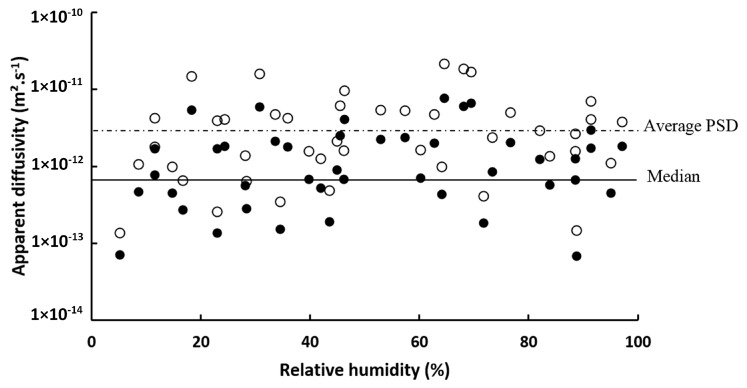
Water vapor apparent diffusivity in cellulose particles at different relative humidities, obtained by using median diameter and length (•) or the particle size distribution (PSD) with median values for each classes (o). Lines represent mean values of apparent diffusivity.

From 2D image analysis results and the water vapor sorption kinetic, water vapor apparent diffusivity in cellulose particles was estimated at each RH step of sorption kinetic by considering the size distribution or using global descriptor (median or mean). Figure 6 shows a comparison between water vapor diffusivities in cellulose estimated by using the global median of diameter and length as global descriptor and the ones estimated while using the PSD-median. It is worth noting that a broad scattering of diffusivity values was observed. This was due to the high sensitivity of the QCM equipment (diffusivity values ranging from 7 × 10^−14^ and 8 × 10^−12^ m^2^·s^−1^), which is able to detect a mass of 7 × 10^−7^ µg in the best case [29]. Thus, a small modification of either particle dispersion state on the quartz substrate, particle morphology or sample mass could affect the apparent diffusivity and lead to such scattered results, as previously observed by other authors [30,31,32]. In the case of PSD-median, the distribution was computed by using bin size of 1 µm × 1 µm. No significant impact of relative humidity on apparent diffusivity was observed. Thus, mean apparent diffusivity, within the meaning of temporal mean, was calculated for each run. It led to apparent diffusivity valued of 8.4 × 10^−13^ ± 8.6 × 10^−13^ m^2^·s^−1^ and 3.1 × 10^−12^ ± 2.3 × 10^−12^ m^2^·s^−1^ for global descriptor and size distribution, respectively, with a relative error of 200% between these two values. In addition, the use of the size distribution reduced the RMSE from 0.01 to 0.005 g·g^−1^, i.e., increasing the fit quality between simulated and experimental water uptakes. Using size distribution allowed for refining results by obtaining a better simulation fit and considering the polydispersity of the sample.

**Figure 7:**
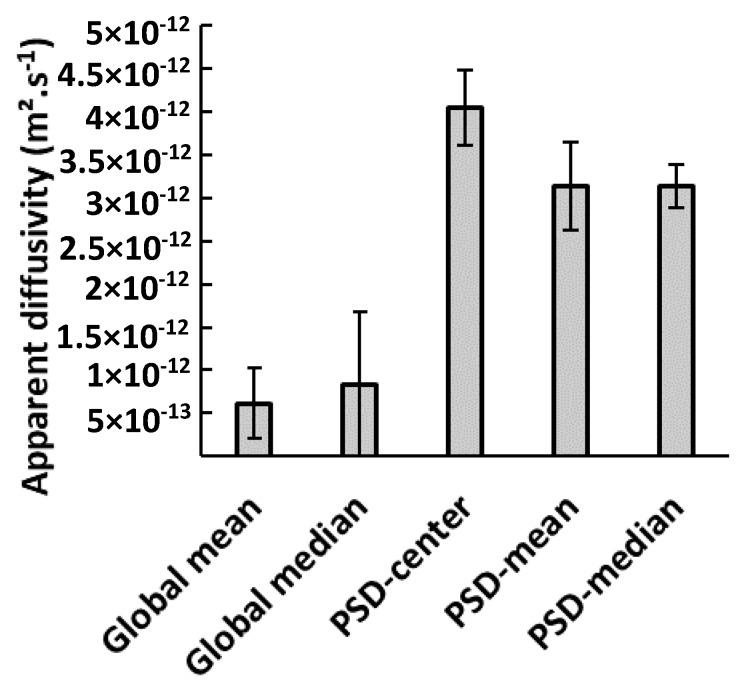
Apparent water vapor diffusivity in cellulose particles estimated for different shape descriptors (bin class of 10 µm × 10 µm).

**Impact of shape descriptors.** The use of different shape descriptors for estimation of diffusivity was investigated: median or mean morphological parameters as global descriptors and 2D size distributions by using the center, mean, or median to obtain representative parameters for each class. Figure 7 presents mean values of apparent diffusivity estimated for the previously listed size descriptors, with squared meshes of 10 µm for the three PSD cases. The main result is that taking into account the PSD, whatever the option considered to represent the particles of a same class (center, mean, or median), systematically leads to higher diffusivities than when using global descriptors. Mean apparent diffusivities in PSD-mean and PSD-median cases were similar, with values close to 3 × 10^−12^ ± 1 × 10^−12^ m^2^·s^−1^ in both cases, while PSD-center led to overestimated values. This latter shape descriptor should be thus excluded, even if was technically the easiest way to integrate the 2D size distribution of the population. This discrepancy is mainly due to the absence of physical meaning of this shape descriptor. Indeed, this indicator is not impacted by the size distribution inside a given class but only by the mesh size of this class. At the opposite, median and mean size descriptors in each class are more representative of the population of particles in this class. Apparent diffusivities that were estimated using global median or mean size descriptors gave, respectively, values of 8.4 × 10^−13^ ± 8.6 × 10^−13^ and 0.9 × 10^−12^ ± 1 × 10^−12^ m^2^·s^−1^. The lower value that was obtained by using mean diameter and length is explained by the fact that these mean parameters are lower than the median ones. Indeed, the equivalent mean cylinder particle is smaller than the equivalent median ones, which implies a lower diffusivity in the mean case to fit the experimental sorption kinetics. Finally, for the considered cellulose particles, the use of global descriptors instead of 2D size distribution, with squared meshes of 10 µm, caused an under-estimation of diffusivity by a factor 3 in the worst case, i.e., global mean versus PSD-mean or PSD-median, PSD-center being discarded due to its lack of physical meaning.

**Figure 8:**
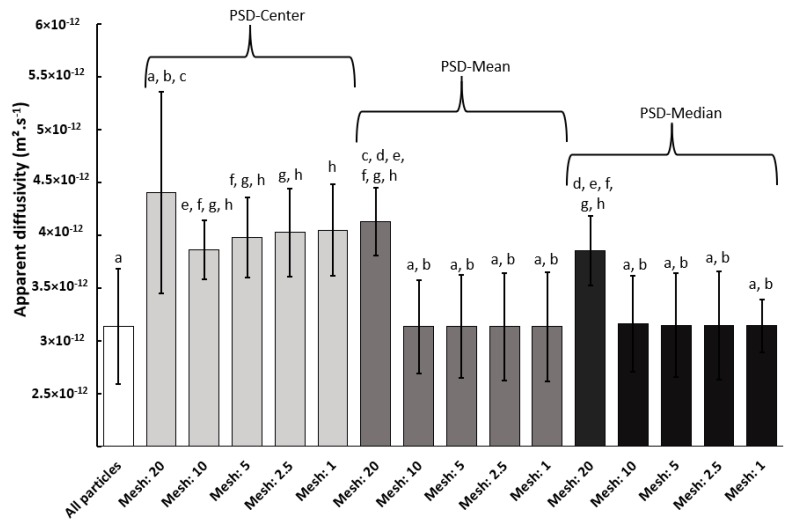
Mean values of apparent diffusivity for different shape descriptors and size meshes. Similar letters in indicate similar means (*p* > 0.05).

**Impact of the class size.** Five different squared meshes were tested, with length of: 20 µm, 10 µm, 5 µm, 2.5 µm, and 1 µm. Figure 8 shows results for these five different class size and for three shape descriptors: PSD-center, PSD-mean, and PSD-median. The use of a coarse mesh (20 µm) led to the same trend, regardless the shape descriptors, and the highest diffusivity. For example, with the PSD-mean, estimated diffusivity was 4 × 10^−12^ m^2^·s^−1^ with a mesh of 20 µm against 3 × 10^−12^ m^2^·s^−1^ with a mesh of 1 µm. The reduction of the mesh size rapidly stabilized the estimated diffusivities around 3 × 10^−12^ m^2^·s^−1^ for PSD-mean and PSD-median, with no significant difference between mesh of 10 µm and 1 µm. Contrariwise, in the PSD-center case, an overestimation of diffusivity was obtained, regardless the size mesh, as already presented in the previous section. This shape descriptor was not adapted to estimate diffusivity of polydisperse cellulose particle as it is only related to the class dimension and not to the population of particles in this class. Indeed, at the most precise case, i.e., when each class contains a single particle, the mean and median gave precisely the diameter and length of this particle, while the center still led to an error on the size parameters.

In order to test the accuracy of these values, the mean diffusivities were also estimated by considering the whole population of particles, corresponding to the use of Equation (10) where each class contains a single particle of the population. The resulting value was 3 × 10^−12^ m^2^·s^−1^, which confirmed the validity of PSD-mean and PSD-median methods and confirmed that PSD-center was not suitable. This estimated diffusivity was in a range of diffusivity values estimated for cellulosic paper, i.e., from 1 × 10^−12^ to 7 × 10^−12^ m^2^·s^−1^ [33]. Difference can be explained by the macrostructure of the sample, i.e., network of cellulose particles (paper) or individual cellulose particles (powder), the crystallinity of cellulose, and the purity of cellulose.

Statistical analysis of variance showed that, for bin size that was lower or equal to 10 µm, the results obtained by considering all the particles, or by using PSD-mean or PSD-median method are in the same class, and significantly different than the results that were obtained by PSD-center approach. Diffusivity estimated by PSD-center method with a bin size of 20 µm showed a large standard deviation and so was not statistically different than the diffusivity estimated by considering all particles. This large standard deviation was due to the stronger deleterious effect of considering the center values of geometrical parameters for this bin size. Finally, apply a bin size lower or equal to 10 µm with PSD-mean of PSD-median approaches allowed for obtaining the most accurate and representative of the sample, estimated vapor diffusivity.

For the studied kind of particles with length lower than 35 µm and diameter lower than 25 µm, the class size of 10 µm was sufficient for accurately estimating the water vapor apparent diffusivity. The more the class size was small, the more the estimation was exact, until reaching an asymptotic value as shown in Table 1. However, it should be noted that the computation time increased when reducing the mesh size. As an example, splitting the mesh by a factor 2 by reducing the class size from 20 µm to 10 µm led to an augmentation of the calculation time by a factor 3. The ultimate solution will be to take into account the geometry of each particle in the model. This method was tested and presented the same value of diffusivity as the one that was obtained for a mesh of 10 µm for PSD-mean and PSD-median while this solution increased extremely computing time (420 s against 80 s) without necessary providing more specific results. Computing time with quad core i7-4810MQ processor was different according to using 2D size distribution or global descriptor, indeed, computing times were around 300 s and less than 80 s for size distribution and global descriptor, respectively. This result was obtained for small number particles around 2000 particles. If the number of particles is very consequent, computing time will have a significant impact. According to the precision of desired results and the available calculation time, it is possible to modulate the choice of shape descriptor by using a global descriptor (preferably the median) or 2D size distribution (center, median or mean). However, in the case where precision is an important choice, it is better to use either the 2D median or 2D mean size distribution.

## 4. Conclusions

Water vapor sorption kinetics in the cellulose particles were recorded at 25 °C by using a quartz crystal microbalance coupled with an adsorption system. Analytical solution of diffusion in a finite cylinder was adapted to diffusion in a heterogeneous population of cylinder particles. For each kinetics, water vapor apparent diffusivity was estimated. In the present work, different shape descriptors, as evaluated by image analysis, have been investigated to estimate apparent diffusivity. The influence of considering a 2D size distribution instead of a single value of shape descriptor was also investigated. Results revealed that the use of an analytical approach when considering the 2D mean-PSD or median-PSD to estimate water vapor apparent diffusivity was the most accurate way to get data at the scale of particle in a polydisperse sample of cellulose particles. Following this approach, a water vapor apparent diffusivity of 3.1 × 10^−12^ ± 2.3 × 10^−12^ m^2^·s^−1^ was found for the considered cellulose sample. Not applying size distribution led to an underestimation of a factor 2. This procedure could be extended for all polydisperse samples in order to have a good characterization of estimated properties at the scale of single particles.

## Figures and Tables

**Table 1:** Water vapor diffusivity for different shape descriptors and meshes.

	Diffusivity (×10^−12^ m^2^·s^−1^ )
Median	0.8 ± 1.0
All particles	3.1 ± 2.3
Center, mesh 20	4.4 ± 2.9
Mean: mesh 20	4.1 ± 2.3
Median: mesh 20	3.8 ± 2.3
Center, mesh 10	3.9 ± 3.3
Mean: mesh 10	3.1 ± 2.4
Median: mesh 10	3.2 ± 2.4
